# Profiling of innate and adaptive immune cells during influenza virus infection reveals sex bias in invariant natural killer T (iNKT) cells

**DOI:** 10.1002/iid3.837

**Published:** 2023-04-12

**Authors:** Piotr Humeniuk, Aidan Barrett, Hannes Axelsson, Carmen Corciulo, Christina Drevinge, Alicia Del Carpio Pons, Davide Angeletti, Julia M. Scheffler, Ulrika Islander

**Affiliations:** ^1^ Department of Rheumatology and Inflammation Research, Institute of Medicine, Sahlgrenska Academy University of Gothenburg Gothenburg Sweden; ^2^ Department of Microbiology and Immunology, Institute of Biomedicine, Sahlgrenska Academy University of Gothenburg Gothenburg Sweden

**Keywords:** airway inflammation, infection, influenza A virus, invariant natural killer T (iNKT) cells, lymphocytes, myeloid cells, sex hormones

## Abstract

**Background:**

Influenza A virus (IAV) infection leads to significant morbidity and mortality. Biological sex influences the immune responses to IAV infection, resulting in higher mortality in women of reproductive age. Previous studies revealed increased activation of T and B cells in female mice after IAV infection, but extensive analysis of sex differences in both innate and adaptive immune cells over time is lacking. Invariant natural killer T (iNKT) cells are fast‐reacting forces and modulators of immune responses that are important to IAV immunity, but it is not known if the presence and function of iNKT cells differ between females and males. The aim of this study was to determine immunological mechanisms that contribute to the increased disease severity in female mice during IAV infection.

**Methods:**

Female and male mice were infected with mouse‐adapted IAV and monitored for weight loss and survival. Immune cell populations and cytokine expression in bronchoalveolar lavage fluid, lung, and mediastinal lymph node were determined at three time points after infection using flow cytometry and ELISA.

**Results:**

The results reveal increased severity and mortality in adult female mice compared to age‐matched males. Female mice show larger increases in innate and adaptive immune cell populations and cytokine production in lung compared to mock on Day 6 postinfection. On Day 9 postinfection, female mice express higher numbers of iNKT cells in lung and liver compared to males.

**Conclusions:**

This comprehensive analysis of immune cells and cytokines over time following IAV infection reveals increased leukocyte expansion and stronger proinflammatory cytokine responses in female mice during disease initiation. Furthermore, this is the first study to report a sex bias in iNKT cell populations after IAV infection. The data suggests that the process of recovery from IAV‐induced airway inflammation is associated with increased expansion of several different iNKT cell subpopulations in female mice.

## Highlights

- Infection with influenza A virus (IAV) leads to increased weight loss and mortality in female mice.
- IAV infection induces increased leukocyte expansion and stronger proinflammatory cytokine responses in the lung of female mice compared to mock on Day 6 postinfection.
- IAV infection leads to a sex‐specific increase of invariant natural killer T (iNKT) cell populations in lung and liver of female mice on Day 9 postinfection.
- The association between weight gain and the sex‐specific increase in iNKT cells on Day 9 postinfection implicate a role for iNKT cells in the resolution of inflammation in female mice.

## Graphical abstract

In this study, comprehensive profiling of innate and adaptive immune cells following influenza A virus infection over time in adult female and male mice was performed. The data reveals higher numbers of lung leukocytes and increased cytokine production in female mice at the onset of symptom progression. Additionally, female mice show a sex‐dependent expansion of invariant natural killer T cell populations in lung at the start of disease recovery.

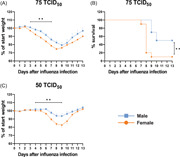

## 1 INTRODUCTION

Influenza is an infectious disease caused by negative‐sense, single‐stranded RNA influenza viruses. Influenza A virus (IAV) is a major public health threat, which causes influenza disease outbreaks with significant suffering and mortality.^1^ Sex hormones influence immune responses, leading to higher mortality in women of reproductive age following exposure to pathogenic IAV strains.^2^, ^3^ The human sex difference in influenza severity can be replicated using mouse models where IAV infection causes increased disease severity and mortality in sexually mature female mice compared to males, and removal of the gonads in both sexes eliminated the observed difference.^4^ It has been shown that the viral titers in lungs did not differ between males and females after IAV infection, whereas higher induction of inflammatory cytokines including IL‐6, IFN‐γ, and TNF‐α was observed in female mice.^4^ In addition, IAV infection has been shown to induce more robust T cell responses^5^, ^6^ and increased B cell activation followed by specific antibody production in females compared to males.^7^ Also, the repair of damaged pulmonary tissue, for example orchestrated by macrophages, is slower in females than in males following IAV infection.^5^ However, comprehensive analysis of sex differences in innate and adaptive immune cells and cytokines over time following IAV infection is lacking.

More recently, other cell types have been implicated in contributing to viral disease pathology. Invariant NKT (iNKT) cells are a CD1d restricted nonclassical T lymphocyte subset that bridges innate and adaptive immune responses.^8^, ^9^ The highest frequency of iNKT cells in mice is found in liver, where they account for around 40% of the intrahepatic lymphocyte population, while they represent around 5% of the resident lymphocytes in lung.^9^ Following infections, iNKT cells are classified as fast reacting forces, modulators of immune responses and efficient cytokine producers.^10^ The importance of iNKT cells for IAV immunity has been confirmed in mouse models. An accelerated rate of weight loss and mortality was observed in iNKT‐deficient mice compared to wild‐type controls.^11^, ^12^ However, no studies have evaluated the importance of iNKT cells in the context of increased pathology in females following IAV infection.

The aim of this study was to determine sex differences in innate and adaptive immune cell populations, and in antiviral and proinflammatory cytokine production, at different time‐points following IAV infection. We found increased disease severity and mortality in sexually mature female mice compared to males, confirming the results from previous studies. Female mice show more severe inflammatory responses on Day 6 postinfection, characterized by larger increases in lung leukocytes, including macrophages, T cells, and B cells, as well as elevated levels of proinflammatory cytokines. Disease recovery, defined as the reversal of weight loss, started on Day 9 post‐IAV infection for both females and males. Female mice display significantly higher numbers of iNKT cell populations in lung and liver on Day 9 postinfection compared to males. The observed sex difference in iNKT cell response suggests involvement of this cell type in the process of recovery after IAV infection, but more research is required to fully understand the underlying mechanism.

## 2 METHODS

### 2.1 Animals

Sexually mature (8 weeks old) female and male C57BL/6J mice were purchased from Charles River Laboratories. Animals were kept in a well controlled, pathogen‐free animal housing facility under regular lighting conditions (12 h light/dark cycles) and had free access to tap water and regular chow. All the experimental procedures were performed in accordance with the ethical permit protocols approved by the Regional Ethical Review Board in Gothenburg, Sweden (Dnr: 2585/2019 and 2230/2019), the 2010/63/EU directive, and ARRIVE guidelines.

### 2.2 Virus infection

Mice were anesthetized with isoflurane and inoculated intranasally (i.n.) with mouse‐adapted A/Puerto Rico/8/1934 H1N1 influenza virus (IAV) (provided by D. Angeletti) diluted in 25 µL Hank's Balanced Salt Solution (HBSS) (Gibco) supplemented with 0.1% bovine serum albumin (BSA) (Sigma). Two doses of virus, 50 and 75 TCID_50_, were used. Mock infection with 25 µL of HBSS + 0.1% BSA was used as a control. Weights of the infected animals were checked every day as a marker of disease severity. Animals infected with the high viral dose 75 TCID_50_ were killed when the weight loss exceeded 25% (according to the ethic Dnr: 2230/2019). Animals infected with the sublethal viral dose 50 TCID_50_ were killed when the weight loss exceeded 15% (according to the ethic Dnr: 2585/2019).

### 2.3 Termination of experiments and samples collection

Mice were anesthetized with a mixture of ketamine (Richter Pharma)/dexmedetomidine hydrochloride (Orion Pharma) and euthanized by exsanguination followed by cervical dislocation. Peripheral blood was collected in 500 µL tubes containing serum gel with a clotting activator (Microvette 500 Z‐Gel; Sarstedt). Serum was extracted and stored at −80°C until use. Lung lavage was performed twice with 250 µL of ice‐cold PBS to collect bronchoalveolar lavage fluid (BALF). The liver was perfused by injection of 10 mL of PBS (room temperature) into the portal vein and the gall bladder was removed. Lungs, mediastinal lymph nodes (MLN), and livers were dissected, and weights were noted.

### 2.4 Cell preparation from dissected tissues

Lungs were minced into small pieces and placed into 3 mL of digestion buffer containing RPMI 1640 medium (without phenol red) (Gibco) supplemented with 5% FBS (Sigma), 25 mM HEPES (Sigma), 1 mg/mL of collagenase type 8 (Sigma), and 0.1 mg/mL of DNase I (Sigma). Digestion was performed at 37°C for 40 min in a shaking water bath. The reaction was stopped by adding 3 mL of stop buffer containing HBSS (Gibco) supplemented with 2% FBS (Sigma) and 2 mM EDTA. Digested lung pieces were pushed through 70 µm cell strainers. Lung leukocytes were isolated using Percoll gradient centrifugation. In brief, lung cells were resuspended in 5 mL of 40% Percoll (Cytiva) and loaded on top of 5 mL of 80% Percoll fraction. After centrifugation at 500*g* in room temperature for 12 min, the interphase containing leukocytes was collected. Cells were pelleted, washed, and resuspended in PBS for further analysis.

Livers were pushed through 70 µm cell strainers. Liver mononuclear cells were isolated using Percoll centrifugation. In brief, liver cells were resuspended in 25 mL of 33.75% percoll (Cytiva) solution and centrifuged at 700*g* in room temperature for 12 min. Pelleted cells were incubated for 6 min in 5 mL of 0.84% ammonium chloride for lysis of red blood cells. The reaction was stopped by the addition of 10 mL of PBS. The remaining cells were washed and resuspended in PBS for further analysis.

MLNs were pushed through 35 µm cell strainer cups on FACS tubes, washed and resuspended in PBS for further analysis. All cells were counted using a hematology analyzer (XP‐300; Sysmex).

### 2.5 Flow cytometry

Two million cells for each sample were stained with eBioscience Fixable Viability Dye eFluor 780 (Thermo Fisher Scientific), followed by incubation with anti‐mouse CD16/CD32 (Fc‐gamma receptor blocking) antibody (2.4G2 RUO; BD Pharmingen). Afterwards, the cells were stained with fluorescence‐labeled antibodies from BioLegend: APC‐CD3 (17A2), Pacific Blue‐CD4 (GK1.5), PE‐Cy7‐CD8a (53‐6.7), BV510‐CD19 (6D5), PE‐Cy7‐F4/80 (BM8), PerCP‐Ly6G (RB6‐8C5), PE‐CD45 (30‐F11), FITC‐CD11c (N418), APC‐CD24 (30‐F1), BV510‐I‐A/I‐E (M5/114.15.2), BV421‐CD11b (M1/70). PE‐Cy7‐PLZF (9E12), PerCP‐Cy5.5‐TCRβ (H57‐597), FITC‐NK1.1 (PK136), BV510‐CD44 (IM7), and Pacific Blue‐CD24 (M1/69); from eBioscience: APC‐RORγt (B2B). For iNKT cell identification mouse CD1d tetramers (PBS‐57 loaded/unloaded, PE‐conjugated) were used. Mouse CD1d tetramers were kindly provided by the NIH Tetramer Core Facility. For transcription factor staining (PLZF and RORγt), an additional staining procedure was performed using Foxp3/Transcription Factor Staining Buffer Set (eBioscience) according to manufacturer instructions. Fluorescence minus one (FMO) stained samples and unstained cells were used as controls. Flow cytometry analyses were performed using a FACSVerse instrument (BD Bioscience). Data were analyzed using FlowJo software (version 10; Tree Star). Invariant NKT cells and their specific subpopulations were defined as described in Supporting Information: Figure S1. Other cell populations were defined as follows: T cells (CD3^+^), CD4+ T cells (CD3,^+^ CD4^+^), CD8+ T cells (CD3,^+^ CD8^+^), B cells (CD19^+^), natural killer (NK) cells (TCRβ,^−^ NK1.1^+^), macrophages (CD45,^+^ Ly6G,^low/medium^ F4/80^+^), neutrophils (CD45,^+^ Ly6G,^high^ CD24^+^), dendritic cells (DC) (CD45,^+^ Ly6G,^low/medium^ F4/80,^−^ CD11c,^high^ CD11b,^high^ I‐A/I‐E^+^).

### 2.6 Determination of cytokines

Cytokine levels in BALF and serum were assessed by ELISA using ELISA MAX^TM^ Deluxe Set Mouse IFN‐α1 (BioLegend), ELISA MAX^TM^ Deluxe Set Mouse TNF‐α (BioLegend), BD OptEIA^TM^ Mouse IFN‐γ ELISA Set (BD Bioscience), DuoSet Mouse IL‐6 (R&D Systems), Invitrogen Mouse IL‐10 Uncoated ELISA (Invitrogen), and Invitrogen Mouse IL‐22 Uncoated ELISA (Invitrogen). All assays were performed according to the manufacturer's instructions.

### 2.7 Statistical analysis

Data is expressed as mean ± standard error of mean. *p* < .05 was considered statistically significant. Animal weight curves were compared using two‐way analysis of variance (ANOVA) for defined periods. Logrank (Mantel−Cox) test were used for comparison of survival rates. Absolute cell counts, cytokine levels, and MHCII expression levels were compared using two‐way ANOVA, with Tukey's multiple comparisons post hoc test to identify differences between the groups. Shapiro−Wilks' normality test was performed to confirm normal distribution, and data that did not meet this criteria was logarithmically transformed before two‐way ANOVA was performed. Differences between female mock‐infected versus female IAV‐infected mice are shown as: # = *p* < .05, ## = *p* < .01, ### = *p* < .001; differences between male mock‐infected versus male IAV‐infected mice are shown as: † = *p* < .05, †† = *p* < .01, ††† = *p* < .001; and differences between female IAV‐infected versus male IAV‐infected mice are shown as: * = *p* < .05, ** = *p* < .01, *** = *p* < .001.

## 3 RESULTS

### 3.1 Infection with IAV leads to more severe disease in female mice

**Figure 1:**
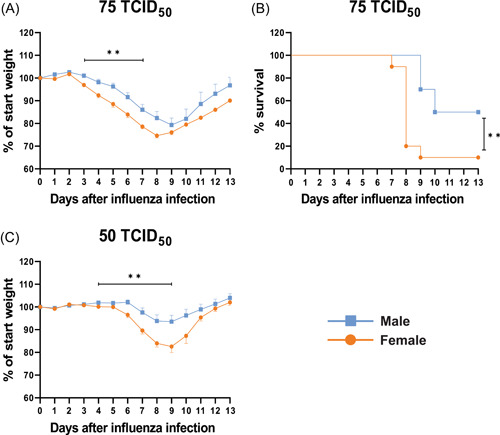
Infection with influenza A virus (IAV) leads to more severe disease in female mice. Female and male C57BL/6J mice (8 weeks old) were infected intranasally (i.n.) with two different doses of mouse‐adapted IAV PR8 H1N1. Mice infected with 75 TCID_50_ were monitored for (A) weight loss and (B) survival. Mice infected with 50 TCID_50_ were monitored for (C) weight loss. *n* = 10/group. (A, C) Two‐way analysis of variance (ANOVA) for defined periods, (B) Logrank (Mantel−Cox) test, **p < .01.

To test whether infection with IAV leads to more severe disease in females, C57BL/6J mice were infected with two different doses of IAV (mouse adapted A/Puerto Rico/8/1934 H1N1). Infection with high dose (75 TCID_50_) highlighted the differences between biological sexes regarding both weight loss and survival (Figure 1A,B). All infected animals started to lose weight at Day 3 postinfection. From Day 3 until Day 7, female mice showed a higher percentage of weight loss compared to males (Figure 1A). The survival rate was lower in females with the biggest difference (80%) observed on Day 8 (Figure 1B). With the lower dose (50 TCID_50_), the first sign of weight loss was observed on Day 6 in female mice and on Day 7 in males. Female mice showed a higher percentage of weight loss between Days 4 and 9 compared to males (Figure 1C). After Day 9 both groups started to recover, and the weights increased above the starting point on Day 13. No difference in the survival rate between female and male mice was observed with the lower IAV dose (data not shown). The lower dose, 50 TCID_50_, was used for all further experiments as it was a sublethal dose where the sex difference in weight loss was preserved.

### 3.2 Increased numbers of total leukocytes and macrophages in lung of female mice on Day 6 post‐IAV infection

**Figure 2:**
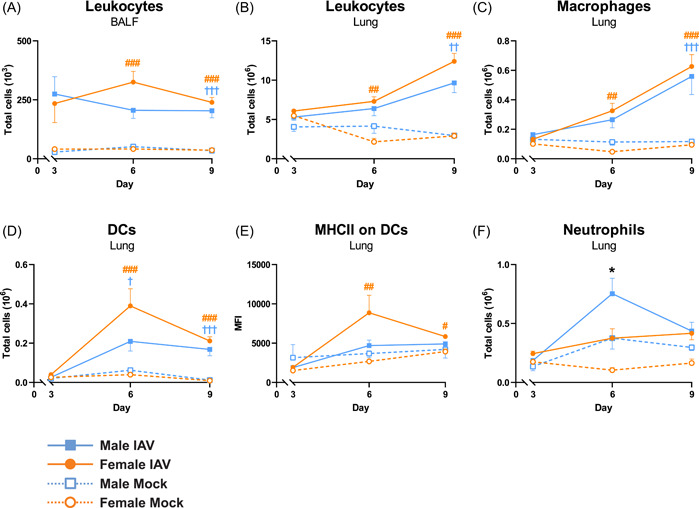
Increased leukocyte infiltration to lung in female mice on Day 6 postinfection. Female and male C57BL/6J mice (8 weeks old) were infected intranasally (i.n.) with 50 TCID_50_ mouse‐adapted influenza A virus (IAV) PR8 H1N1 and killed on Days 3, 6, or 9 postinfection. (A) Total leukocyte numbers in bronchoalveolar lavage fluid (BALF) were analyzed by manual microscopic counting. Lung tissues were digested to obtain single cell suspensions and analyzed for (B) total leukocyte numbers by automatic cell counting, and by flow cytometry for (C) macrophages, (D) dendritic cells (DCs), (E) MHC class II expression on DCs expressed as geometric mean fluorescent intensity (MFI), and (F) neutrophils. Data is expressed as mean ± standard error of mean (SEM), *n* = 5 for mock, *n* = 10 for IAV. Two‐way ANOVA followed by Tukey's multiple comparison test, female mock‐infected versus female IAV‐infected #*p* < .05, ##*p* < .01, ###*p* < .001; male mock‐infected versus male IAV‐infected ^†^ *p* < .05, ^††^ *p* < .01, ^†††^ *p* < .001; female IAV‐infected versus male IAV‐infected *p < .05. ANOVA, analysis of variance.

Female and male mice were infected with mock or 50 TCID_50_ of mouse‐adapted PR8 H1N1 IAV and killed on Days 3, 6, and 9 postinfection. On Day 3 postinfection, no significant differences between the groups were found for total leukocytes in BALF or lung tissue (Figure 2A,B), or for any of the analyzed myeloid cell populations in lung tissue (Figure 2C,D,F). On Day 6 post‐IAV infection, only female mice showed a significant increase in total leukocyte numbers in BALF and lung tissue compared to mock‐infected controls, although infected male mice showed a trend toward increased leukocyte numbers in BALF (Figure 2A,B). On Day 9 post‐IAV infection, both female and male mice had significantly increased numbers of leukocytes in BALF and lung tissue compared to their respective mock‐infected controls (Figure 2A,B). A similar pattern was observed for total number of macrophages isolated from lung tissue (Figure 2C). DC were significantly increased in both female and male infected mice compared to mock on Days 6 and 9 (Figure 2D) The expression level of MHC class II on DCs did not differ between infected male mice and mock on Days 3, 6, or 9, whereas female mice showed significantly increased levels compared to mock on Days 6 and 9 postinfection (Figure 2E). The number of lung neutrophils was significantly higher in infected males compared to infected females on Day 6 postinfection (Figure 2F).

### 3.3 Increased numbers of T cells in MLN of female mice on Day 6 postinfection

**Figure 3:**
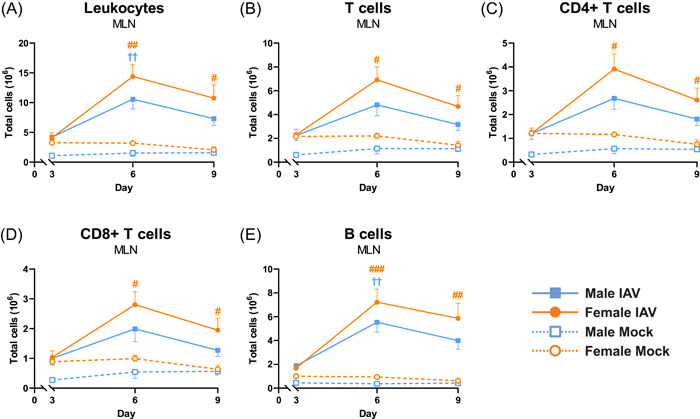
T cell populations in mediastinal lymph node (MLN) increase in females on Day 6 postinfection. Female and male C57BL/6J mice (8 weeks old) were infected intranasally (i.n.) with 50 TCID_50_ mouse‐adapted influenza A virus (IAV) PR8 H1N1 and killed on Days 3, 6, or 9 postinfection. Single‐cell suspensions from MLN were analyzed for (A) total leukocyte numbers by automatic cell counting, and by flow cytometry for (B) total T cells, (C) CD4+ T cells, (D) CD8+ T cells, (E) B cells. Data is expressed as mean ± standard error of mean (SEM), *n* = 5 for mock, *n* = 10 for IAV. Two‐way ANOVA followed by Tukey's multiple comparison test, female mock‐infected versus female IAV‐infected #*p* < .05, ##*p* < .01, ###*p* < .001; male mock‐infected versus male IAV‐infected ^††^ *p* < .01. ANOVA, analysis of variance.

On Day 3 postinfection, no significant differences between the groups were found for total leukocytes, T cell populations or B cells in MLN (Figure 3A−E). On Day 6 postinfection, the total number of leukocytes in MLN was increased in both female and male mice compared to mock (Figure 3A). On Day 9 postinfection, only female mice had significantly increased leukocyte numbers, although a trend toward an increase was shown also in males (Figure 3A). The numbers of total T cells, CD4+ T cells and CD8+ T cells in MLN were increased in females compared to mock on Days 6 and 9 postinfection (Figure 3B−D). The number of B cells in MLN was increased compared to mock in both female and male mice on Day 6, but only in females on Day 9 post‐IAV infection (Figure 3E).

### 3.4 Increased numbers of T cells and B cells in lung of female mice on Day 6 postinfection

**Figure 4:**
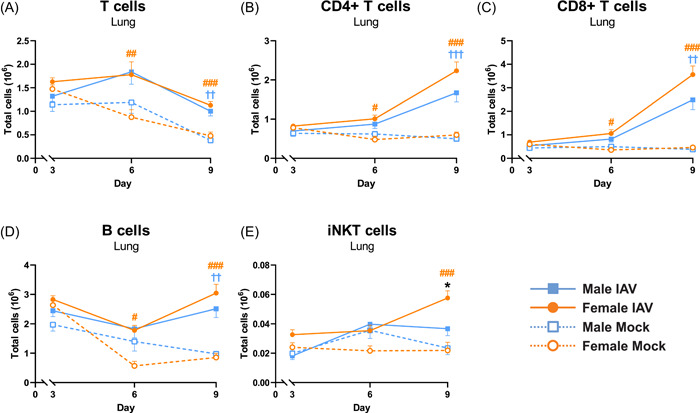
The number of iNKT cells in lung is significantly higher in females compared to males on Day 9 postinfection. Female and male C57BL/6J mice (8 weeks old) were infected intranasally (i.n.) with 50 TCID_50_ mouse‐adapted influenza A virus (IAV) PR8 H1N1 and killed on Days 3, 6, or 9 postinfection. Lungs were digested to obtain single‐cell suspensions and analyzed by flow cytometry for (A) total T cells, (B) CD4+ T cells, (C) CD8+ T cells, (D) B cells, and (E) total iNKT cells. Data is expressed as mean ± standard error of mean (SEM), *n* = 5 for mock, *n* = 10 for IAV. Two‐way ANOVA followed by Tukey's multiple comparison test, female mock‐infected versus female IAV‐infected #*p* < .05, ##*p* < .01, ###*p* < .001; male mock‐infected versus male IAV‐infected ^††^ *p* < .01; ^†††^ *p* < .001; female IAV‐infected versus male IAV‐infected *p < .05. ANOVA, analysis of variance; iNKT, invariant natural killer T.

On Day 3 postinfection, no significant differences between the groups were found for any of the analyzed T cells populations (Figure 4A−C) or B cells in lung tissue (Figure 4D). On Day 6 postinfection, a significant increase in total T cells, CD4+ T cells, CD8+ T cells, and B cells was observed in lung of infected female, but not male mice, compared to mock. On Day 9, these cell populations were significantly increased in both female and male IAV‐infected mice compared to mock (Figure 4A−D).

### 3.5 The number of iNKT cells in lung and liver is significantly higher in females compared to males on Day 9 postinfection

Flow cytometry analysis of iNKT cells in lung tissue revealed no significant differences between the groups on Days 3 and 6 postinfection (Figure 4E). On Day 9 post‐IAV infection female mice had significantly increased numbers of iNKT cells in lung compared to mock and to male IAV‐infected mice (Figure 4E). The observed sex difference in iNKT cells on Day 9 was also confirmed in liver (Supporting Information: Figure S2).

### 3.6 Sex‐specific increase of iNKT cell populations in lung of female mice on Day 9 post‐IAV infection

**Figure 5:**
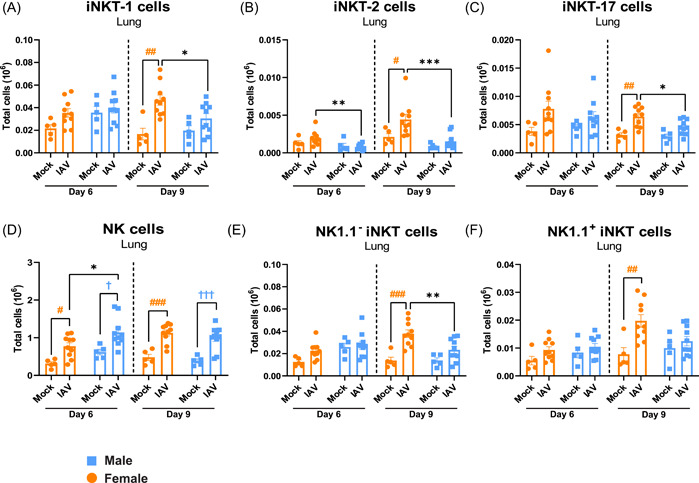
Sex‐specific increase of iNKT cell subpopulations in lung of female mice on Day 9 postinfection. Female and male C57BL/6J mice (8 weeks old) were infected intranasally (i.n.) with 50 TCID_50_ mouse‐adapted influenza A virus (IAV) PR8 H1N1 and killed on Days 6 or 9 postinfection. Lungs were digested to obtain single‐cell suspensions and analyzed by flow cytometry for (A) iNKT‐1 cells, (B) iNKT‐2 cells, (C) iNKT‐17 cells, (D) NK cells, (E) NK1.1^−^ iNKT cells, and (F) NK1.1^+^ iNKT cells. Data is expressed as mean ± standard error of mean (SEM), *n* = 5 for mock, *n* = 10 for IAV. Two‐way ANOVA followed by Tukey's multiple comparison test, female mock‐infected versus female IAV‐infected #*p* < .05, ##*p* < .01, ###*p* < .001; male mock‐infected versus male IAV‐infected ^†^ *p* < .05, ^†††^ *p* < .001; female IAV‐infected versus male IAV‐infected *p < .05, **p < .01, ***p < .001. ANOVA, analysis of variance; iNKT, invariant natural killer T.

To investigate the sex difference in iNKT cells during IAV infection more thoroughly, detailed flow cytometry analysis of iNKT cell subpopulations as well as NK cells in lung tissue was performed on Days 6 and 9 post‐IAV infection (gating strategy of iNKT cell populations is shown in Supporting Information: Figure S1). On Day 9 postinfection, a sex‐specific increase in the subpopulations iNKT‐1, iNKT‐2, and iNKT‐17 was observed in female mice (Figure 5A−C), and for iNKT‐2 cells the sex specific increase in infected female mice compared to infected male mice was shown already on Day 6 postinfection (Figure 5B). NK cells were significantly increased in infected mice of both sexes compared to mock on Days 6 and 9, and male infected mice had significantly higher numbers of NK cells compared to female infected mice on Day 6 post‐IAV infection (Figure 5D). NK1.1^−^ iNKT cells were significantly increased in female infected mice on Day 9 compared to both mock and male infected mice (Figure 5E). Also, a significant increase of NK1.1^+^ iNKT cells was observed in infected female but not male mice compared to mock on Day 9 post‐IAV infection (Figure 5F).

### 3.7 Elevated cytokine responses in female mice on Day 6 post‐IAV infection

**Figure 6:**
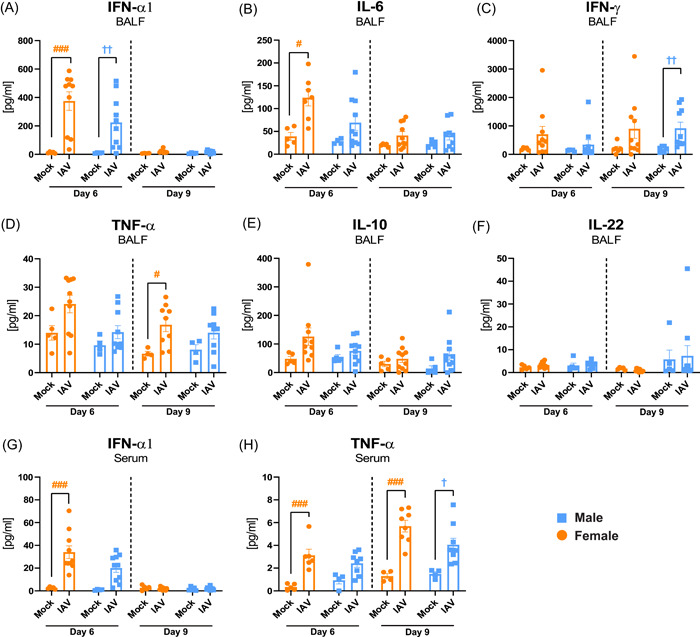
Elevated antiviral and proinflammatory cytokine responses in female mice on Day 6 postinfection. Female and male C57BL/6J mice (8 weeks old) were infected intranasally (i.n.) with 50 TCID_50_ mouse‐adapted influenza A virus (IAV) PR8 H1N1 and killed on Days 6 or 9 postinfection. (A−F) Bronchoalveolar lavage fluid (BALF) was collected and analyzed by ELISA for levels of (A) IFN‐α1, (B) IL‐6, (C) IFN‐γ, (D) TNF‐α, (E) IL‐10, and (F) IL‐22. Serum was analyzed by ELISA for (G) IFN‐α1 and (H) TNF‐α levels. Data is expressed as mean ± standard error of mean (SEM), *n* = 4−5 for mock, *n* = 6−10 for IAV. Two‐way ANOVA followed by Tukey's multiple comparison test, female mock‐infected versus female IAV‐infected #*p* < .05, ###*p* < .001; male mock‐infected versus male IAV‐infected ^†^ *p* < .05, ^††^ *p* < .01. ANOVA, analysis of variance.

In addition to iNKT cell analysis, cytokine levels in BALF and serum were investigated by ELISA on Days 6 and 9 postinfection. Infected female and male mice showed a significant increase in IFN‐α1 levels in BALF on Day 6 compared to mock, whereas only female mice showed an increase in IL‐6 levels (Figure 6A,B). The levels of IFN‐γ in BALF were increased in infected male mice, whereas TNF‐α in BALF was increased in infected female mice, compared to mock on Day 9 postinfection (Figure 6C,D). Levels of IL‐10 and IL‐22 measured in BALF were not changed for either sex on Days 6 and 9 post‐IAV infection compared to mock (Figure 6E,F). The cytokine responses were also reflected in serum where IFN‐α1 and TNF‐α levels were increased in IAV infected female mice compared to mock on Day 6 postinfection (Figure 6G,H). On Day 9 postinfection, both female and male mice had significantly increased serum levels of TNF‐α (Figure 6H).

## 4 DISCUSSION

The results from this study show more severe disease development in female mice subjected to IAV infection compared to males. This associates with larger increases in lung leukocytes, and elevated levels of proinflammatory cytokines at the start of disease progression. Female mice also show increased expansion of several different iNKT cell subpopulations in lung and liver at the late stage of infection, coinciding with the start of disease recovery. This novel finding could provide a potential new avenue of exploration for the understanding of mechanisms underpinning sex differences in IAV infection severity.

Men and women differ in immune responses to IAV infections.^3^ During childhood, boys display an increased risk for severe IAV infection compared to age matched girls.^13^, ^14^ The same applies to the elderly, among which aged men have a higher risk of hospitalization compared to elderly women.^15^ However, the pattern is reversed in the reproductive age, when women have a higher incidence of severe disease.^14^ Previous studies have shown that both T cells and B cells, as well as the production of proinflammatory cytokines including IL‐6, IFN‐γ, and TNF‐α are more increased in females than in male mice after infection with IAV.^4^, ^5^, ^6^, ^7^ However, extensive analysis of sex differences in immune cells and cytokine production following IAV infection over time is lacking.

The aim of this study was to comprehensively profile innate and adaptive immune cell populations and cytokine production from the initiation to the resolution of airway inflammation induced by IAV infection in adult female and male mice, to elucidate mechanisms contributing to the observed sex differences. We confirm previously published reports showing that female mice display more severe IAV infection compared to males.^4^ A clear difference in weight loss between sexes was observed with both lethal and sublethal doses of the PR8 IAV strain. Also, increased mortality was shown in female mice using the higher virus dose. Immune cells were investigated in detail at three defined time points post‐IAV infection (Days 3, 6, and 9). Day 3 represents an early time point in which disease symptoms were not yet detectable and no differences in innate and adaptive immune cells was found. On Day 6 postinfection, the first clear difference in weight loss between female and male mice was observed. The start of weight loss in infected females associated with increased numbers of total leukocytes in lung tissue, including macrophages, CD4+ T cells, CD8+ T cells, and B cells, compared to mock. The increased number of T cells in females on Day 6 postinfection was also reflected in MLN, the lymph node draining the lung. On Day 9 postinfection both females and males showed increased numbers of total leukocytes in lung including macrophages, DCs, CD4+ T cells, CD8+ T cells, and B cells. Analysis of proinflammatory cytokines in BALF and serum revealed increased levels of IL‐6 and TNF‐α in females on Day 6 postinfection, while on Day 9 postinfection females and males showed more similar responses.

iNKT cells are major producers of IFNγ and have been shown to be important in the protection against IAV infections.^11^, ^12^ iNKT cells are present in lung tissue and their function can be influenced by estrogen through their expression of estrogen receptor alpha (ERα).^16^ Treatment with the primary iNKT cell antigen, α‐Galactosylceramide (α‐GalCer), leads to higher IFN‐γ production in sexually mature female mice compared to age matched males.^16^ However, the influence of iNKT cells on the increased IAV disease severity in women of reproductive age still remains elusive.

In this study, iNKT cells in lung and liver were unaltered in both sexes compared to mock at Days 3 and 6 post‐IAV infection. However, these populations were increased in lung and liver of infected female mice compared to infected males on Day 9 postinfection, the time point with the largest difference in weight loss between sexes. From this time, female mice quickly recovered and male mice also started to return to their initial weight, indicating the resolution of inflammation.

No difference in BALF IFNγ was detected in response to infection on either Days 6 or 9, indicating that this cytokine is not important for the the observed sex difference in IAV disease pathology. It has been shown that iNKT cells can decrease lung immune pathology by reducing the accumulation of inflammatory monocytes in the lungs,^12^ and production of IL‐22 by iNKT cells was shown to prevent the IAV‐triggered cell death of pulmonary epithelium.^17^ However, in this study the levels of IL‐22 measured in BALF on Days 6 and 9 postinfection was not changed compared to mock for either sex.

Several subpopulations of iNKT cells, which mirror the cytokine production profile of differentiated T helper cells, have been identified including: iNKT‐1 (IFN‐γ, IL‐4), iNKT‐2 (IL‐4, IL‐5, IL‐13), and iNKT‐17 (IL‐17) cells.^9^, ^18^ In this study, all three subpopulations were increased in lung of infected female mice compared to infected males on Day 9, reflecting the overall increase in iNKT cells in lung. The association between weight gain and the sex‐specific increase in several different iNKT cell populations implies a role for iNKT cells in the resolution of inflammation in female mice.

iNKT cells can provide both direct (by interaction with CD1d on antigen‐presenting B cells) and indirect (CD4+ T cell and DC‐associated) help for B cells.^19^ During viral infections, iNKT cells need two stimulation signals from antigen presenting cells to become activated: recognition of self‐lipid‐loaded CD1d by their TCR, and stimulation with type one interferon.^10^ In line with this, a significant increase in lung DCs and levels of IFNα‐1 in serum was observed in female mice in this study. Thus, the increased disease pathology in females, starting on Day 6 postinfection, is not initiated by expansion of iNKT cells. Instead, we speculate that the more severe disease progression in females is driven by the increased myeloid and lymphocyte cell infiltration/expansion and associated cytokine responses in the female lung.

A limitation to this study is the lack of mechanistical insight into the possible involvement of iNKT cells on the resolution of the disease, as well as into the specific role of the different iNKT cell subpopulations. Furthermore, the sex specific difference in iNKT cell response to IAV infection must be investigated in more detail to elucidate the immunological mechanisms involved in recovery. As protective cells, it is possible that iNKT cells expand more efficiently in females to block or repair occuring tissue damage during IAV infection. These questions need to be addressed in future studies.

### 4.1 Perspectives and significance

The sex difference in disease severity to IAV infection is well documented in humans as well as in murine models. However, extensive analysis of sex differences in innate and adaptive immune cell populations, followed over time postinfection, has not previously been reported. Furthermore, sex differences in the non‐classical T cell subpopulation iNKT cells during IAV infection have never previously been investigated. This study reveals increased expansion of both myeloid and lymphocytic cell populations, as well as stronger proinflammatory cytokine responses in female mice on Day 6 post‐IAV infection. Furthermore, female mice show a sex dependent expansion of lung and liver iNKT cell populations on Day 9 post‐IAV infection. This implicates the involvement of iNKT cells in the process of disease recovery after IAV infection, but more research is required to fully understand the underlying mechanism.

## AUTHOR CONTRIBUTIONS

Piotr Humeniuk, Julia M. Scheffler, Davide Angeletti, and Ulrika Islander conceived and designed the research. Piotr Humeniuk, Aidan Barrett, Julia M. Scheffler, Carmen Corciulo, Christina Drevinge, Hannes Axelsson, and Alicia Del Carpio Pons performed the experiments. Piotr Humeniuk, Julia M. Scheffler, Davide Angeletti, and Ulrika Islander analyzed the data. Piotr Humeniuk, Julia M. Scheffler, Davide Angeletti, and Ulrika Islander interpreted the results. Piotr Humeniuk, Aidan Barrett, Julia M. Scheffler, and Ulrika Islander prepared the figures. Piotr Humeniuk, Aidan Barrett, Julia M. Scheffler, and Ulrika Islander drafted the manuscript. All authors revised the draft and approved the final version of the manuscript.

## CONFLICT OF INTEREST STATEMENT

The authors declare no conflict of interest.

## ETHICS STATEMENT

All animal experiments in this study were performed in accordance with the ethical permit protocols approved by the Regional Ethical Review Board in Gothenburg, Sweden (Dnr: 2585/2019 and 2230/2019).

## Supporting information


**Supplementary figure 1**.

**Supplementary figure 2**.

## Data Availability

The data that support the findings of this study are available from the corresponding author upon reasonable request.
