# Supplementary figures and images for: Profiling of innate and adaptive immune cells during influenza virus infection reveals sex bias in invariant natural killer T (iNKT) cells
Source: Immun Inflamm Dis. 2023 Apr 12;11(4):e837. doi: 10.1002/iid3.837 (PMC10091374; doi:10.1002/iid3.837)

Supplementary Figure 1

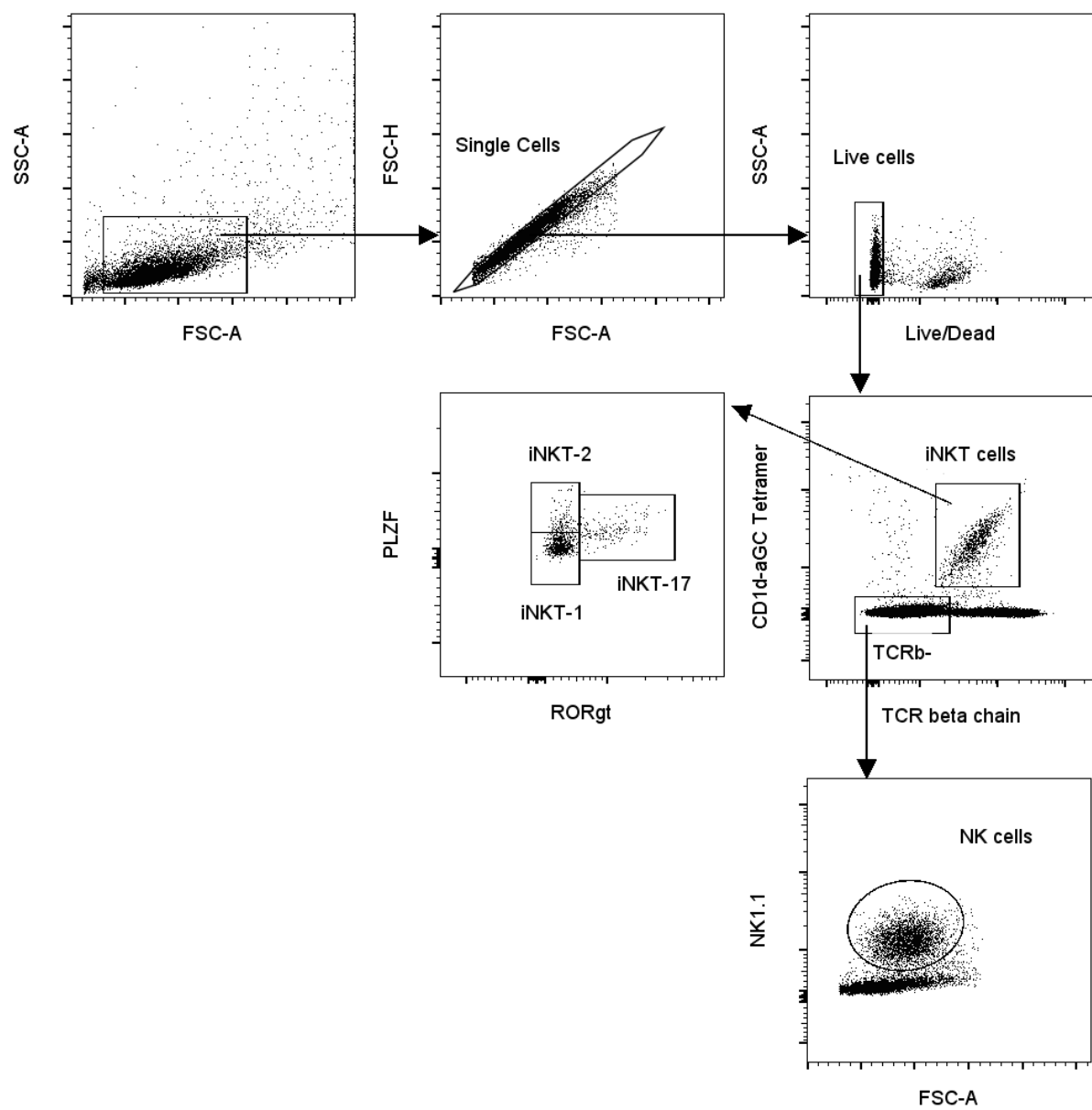

Supplement: Supplementary file 1 — Supplementary figure 1. [file IID3-11-e837-s001.pdf]

Supplementary Figure 2

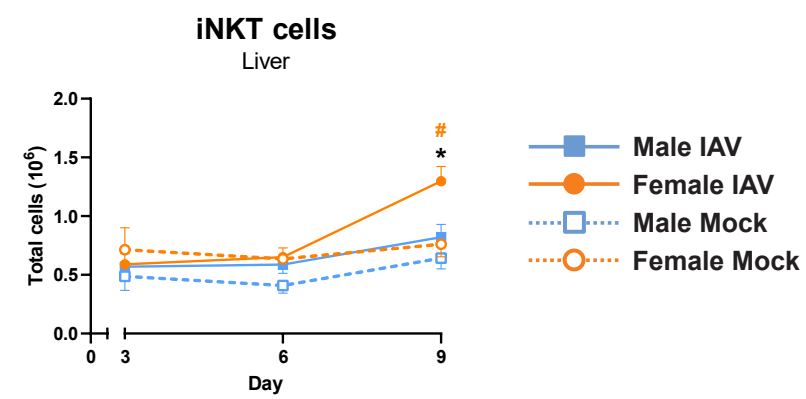

Supplement: Supplementary file 2 — Supplementary figure 2. [file IID3-11-e837-s002.pdf]
